# Phenotypes Associated with Knockouts of Eight Dense Granule Gene Loci (*GRA2-9*) in Virulent *Toxoplasma gondii*

**DOI:** 10.1371/journal.pone.0159306

**Published:** 2016-07-26

**Authors:** Leah M. Rommereim, Valeria Bellini, Barbara A. Fox, Graciane Pètre, Camille Rak, Bastien Touquet, Delphine Aldebert, Jean-François Dubremetz, Marie-France Cesbron-Delauw, Corinne Mercier, David J. Bzik

**Affiliations:** 1 Department of Microbiology and Immunology, The Geisel School of Medicine at Dartmouth, Lebanon, NH, United States of America; 2 Laboratoire Adaptation et Pathogénie des Micro-organismes, Université Grenoble Alpes, Université Joseph Fourier, Grenoble, France; 3 Centre National de la Recherche Scientifique, Unité Mixte de Recherche 5163, Grenoble, France; 4 Station de Cytométrie en Images en Microbiologie (SCIMI platform), Grenoble, France; 5 Université Montpellier 2, Place Eugène Bataillon, Montpellier, France; 6 Centre National de al Recherché Scientifique, Unité Mixte de Recherché 5235, Montpellier, France; University at Buffalo, UNITED STATES

## Abstract

*Toxoplasma gondii* actively invades host cells and establishes a parasitophorous vacuole (PV) that accumulates many proteins secreted by the dense granules (GRA proteins). To date, at least 23 GRA proteins have been reported, though the function(s) of most of these proteins still remains unknown. We targeted gene knockouts at ten *GRA* gene loci (*GRA1-10*) to investigate the cellular roles and essentiality of these classical GRA proteins during acute infection in the virulent type I RH strain. While eight of these genes (*GRA2-9*) were successfully knocked out, targeted knockouts at the *GRA1* and *GRA10* loci were not obtained, suggesting these GRA proteins may be essential. As expected, the Δ*gra2* and Δ*gra6* knockouts failed to form an intravacuolar network (IVN). Surprisingly, Δ*gra7* exhibited hyper-formation of the IVN in both normal and lipid-free growth conditions. No morphological alterations were identified in parasite or PV structures in the Δ*gra3*, Δ*gra4*, Δ*gra5*, Δ*gra8*, *or* Δ*gra9* knockouts. With the exception of the Δ*gra3* and Δ*gra8* knockouts, all of the GRA knockouts exhibited defects in their infection rate *in vitro*. While the single GRA knockouts did not exhibit reduced replication rates *in vitro*, replication rate defects were observed in three double *GRA* knockout strains (Δ*gra4*Δ*gra6*, Δ*gra3*Δ*gra5* and Δ*gra3*Δ*gra7*). However, the virulence of single or double GRA knockout strains in CD1 mice was not affected. Collectively, our results suggest that while the eight individual GRA proteins investigated in this study (GRA2-9) are not essential, several GRA proteins may provide redundant and potentially important functions during acute infection.

## Introduction

*Toxoplasma gondii* is an obligate intracellular protozoan pathogen capable of infecting any living nucleated cell [1]. As one of the most successful protozoan parasites in the group of cyst-forming *Apicomplexa*, *Toxoplasma* is estimated to chronically infect at least a third of the world’s population [2]. Infections in immune-competent individuals are typically asymptomatic, though toxoplasmosis can cause severe pathological effects in immune privileged areas such as the eye or developing fetus [3], and toxoplasmosis is life-threatening in immunocompromised patients [4].

*Toxoplasma* enters host cells via a rapid active invasion mechanism [5] and utilizes the host cell plasma membrane to form, within the host cytosol, a distinct compartment termed the parasitophorous vacuole (PV), in which it replicates and divides [6–8]. *Toxoplasma* invasion and PV formation require three *Apicomplexa*-specific organelles: the micronemes, rhoptries and dense granules. The secreted microneme proteins (MICs) [9] aid in adhesion to the host cell and, with the secreted rhoptry neck proteins (RONs) [10], in formation of a moving junction through which the motile parasite penetrates the host cell. The rhoptry organelles also release rhoptry bulb proteins (ROPs) into the host cell cytosol during invasion. Many ROP proteins re-localize to the non-fusogenic [11] PV membrane (PVM), while other ROP proteins remain in the host cytoplasm or gain access to the host cell nucleus where they contribute to reprograming host gene expression [12].

Shortly after the formation of the PV, the dense granule proteins (GRAs) that are defined by their localization in electron dense granule organelles are massively secreted into the PV lumen [13–17]. A few proteins secreted by the dense granules exhibit homologies to proteins of known function such as cathepsin C [18], nucleoside triphosphate hydrolases (NTPases) [19, 20], an osteopontin-like protein [21], protease inhibitors [22, 23] and a lipolytic lecithin:cholesterol acyltransferase [24]. Yet, most GRA proteins do not have any identifiable gene homologues in species other than cyst-forming coccidians. Within the PV lumen, GRA2 initiates the formation of the membranous tubules that compose the intravacuolar network (IVN) and GRA6 stabilizes this network [25, 26], which is proposed to provide a scaffold for parasite replication [27, 28]. GRA7 aids in the formation of host organelle sequestering tubular structures (HOSTs), which are deep PVM invaginations that entrap single shortened host microtubules to direct host endocytic vacuoles to the PV for nutrient acquisition [29]. GRA7 also interacts with ROP2 and ROP4 [30] and acts in complex with ROP18 to bind host Immunity-related GTPase a6 (Irga6) [31], mediating resistance to a major interferon-γ (IFN-γ) triggered macrophage killing mechanism [32]. GRA22 was recently proposed to play a role in parasite egress [33]. GRA17 and GRA23 were identified as PVM-localized GRAs that mediate passive transport of small molecules [34]. Polymorphic type I GRA6 was recently shown to manipulate the host cell by activating the host transcription factor nuclear factor of activated T cells 4 (NFAT4) [35]. GRA5 increases the expression of CCR7 [36] and GRA25 induces the expression of CCL2 and C-X-C motif ligand 1 (CXCL1) [37]. Other GRA proteins are exported from the PV into the host cell cytosol and/or nucleus where they modify host cell signaling pathways [38]. This exported GRA protein class includes GRA15 [39], GRA16 [40], and GRA24 [41].

Genes encoding several GRA proteins identified in antigen-mapping studies have been previously deleted in virulent type I strains (GRA2, GRA5, GRA6, GRA7, GRA14 and GRA22) [25, 29, 31, 33, 42–44], or in low-virulence type II strains (GRA3, GRA4 and GRA6) [45, 46]. However, previous gene deletion studies in non-Δ*ku80* strains are complicated by frequent off-site mutation that could influence observed phenotypes [47].

In this study, we utilized the virulent type I Δ*ku80* strain that enables highly efficient and precise development of gene knockouts [46–48] or gene tagging [49] to target gene deletions at the first ten *GRA* gene loci (*GRA1-10*). We isolated 8 of the 10-targeted knockouts (Δ*gra2-9*) and investigated invasion, growth, morphology and virulence phenotypes. Overall, our findings validate phenotypes associated with several previously reported *GRA* knockouts, and suggest that while GRA proteins (GRA2-9) are individually not essential, several of these GRA proteins are likely to provide redundant and potentially crucial functions during acute infection.

## Materials and Methods

### Primers

All oligonucleotide primers used in the development of plasmids for targeting gene deletions (S1 Table) and primers used in the validation of targeted gene deletions (S2 Table) are shown in the supplementary material. Sequences for primer design and validation of targeting plasmids were obtained from ToxoDB [www.toxodb.org] [50].

### Plasmid Construction

Plasmids were developed using yeast recombination cloning that fused a ~1-kb 5’ target flank, a ~2-kb hypoxanthine-xanthine-guanine-phosphoribosyltransferase (*HXGPRT*) selectable marker cassette and a ~1-kb 3’ target flank with pRS416 by homologous crossovers at recombination junctions [48]. DNA elements for yeast recombination were amplified from type I RH (EP; ATCC 40050) genomic DNA. All targeting plasmids were validated by restriction enzyme digests and by DNA sequencing. The sequenced and fully annotated type I GT1 strain [www.toxodb.org] was used to identify GRA gene loci and type I sequences for targeting plasmid assembly. Plasmid pΔGRA1 was constructed by fusing the *HXGPRT* minigene cassette between a 1,095-bp 5’ *GRA1* genomic target flank and a 940-bp 3’ *GRA1* genomic target flank to delete nucleotides 5308191 to 5309090 of the *GRA1* locus on chromosome VIII annotated as TGGT1_270250. Plasmid pΔGRA2 was constructed by fusing the *HXGPRT* minigene cassette between a 1,136-bp 5’ *GRA2* genomic target flank and a 1,025-bp 3’ *GRA2* genomic target flank to delete nucleotides 814572 to 812564 of the *GRA2* locus on chromosome X annotated as TGGT1_227620. Plasmid pΔGRA2C was constructed by digesting pΔGRA2 with *Spe*I, followed by self-ligation to remove the *HXGPRT* minigene cassette. Plasmid pΔGRA3 was constructed by fusing the *HXGPRT* minigene cassette between a 950-bp 5’ *GRA3* genomic target flank and a 860-bp 3’ *GRA3* genomic target flank to delete nucleotides 988787 to 989625 of the *GRA3* locus on chromosome X annotated as TGGT1_227280. Plasmid pΔGRA3C was constructed by digesting pΔGRA3 with *Spe*I, followed by self-ligation to remove the *HXGPRT* minigene cassette. Plasmid pΔGRA4 was constructed by fusing the *HXGPRT* minigene cassette between a 1,130-bp 5’ *GRA4* genomic target flank and a 988-bp 3’ *GRA4* genomic target flank to delete nucleotides 1201331 to 1200129 of the *GRA4* locus on chromosome XI annotated as TGGT1_310780. Plasmid pΔGRA4C was constructed by digesting pΔGRA4 with *Spe*I, followed by self-ligation to remove the *HXGPRT* minigene cassette. Plasmid pΔGRA5 was constructed by fusing the *HXGPRT* minigene cassette between a 1,095-bp 5’ *GRA5* genomic target flank and a 956-bp 3’ *GRA5* genomic target flank to delete nucleotides 1753723 to 1754102 of the *GRA5* locus on chromosome V annotated as TGGT1_286450. Plasmid pΔGRA6 was constructed by fusing the *HXGPRT* minigene cassette between a 1,057-bp 5’ *GRA6* genomic target flank and a 975-bp 3’ *GRA6* genomic target flank to delete nucleotides 7195269 to 7194367 of the *GRA6* locus on chromosome X annotated as TGGT1_275440. Plasmid pΔGRA7 was constructed by fusing the *HXGPRT* minigene cassette between a 1,164-bp 5’ *GRA7* genomic target flank and a 954-bp 3’ *GRA7* genomic target flank to delete nucleotides 2582896 to 2585701 of the *GRA7* locus on chromosome VIIa, annotated as TGGT1_203310. Plasmid pΔGRA8 was constructed by fusing the *HXGPRT* minigene cassette between a 1,151-bp 5’ *GRA8* genomic target flank and a 1,015-bp 3’ *GRA8* genomic target flank to delete nucleotides 1894848 to 1895699 of the *GRA8* locus on chromosome III annotated as TGGT1_354720. Plasmid pΔGRA9 was constructed by fusing the *HXGPRT* minigene cassette between a 1,110-bp 5’ *GRA9* genomic target flank and a 971-bp 3’ *GRA9* genomic target flank to delete nucleotides 5508787 to 5510441 of the *GRA9* locus on chromosome XII annotated as TGGT1_251540. Plasmid pΔGRA10 was constructed by fusing the *HXGPRT* minigene cassette between a 1,170-bp 5’ *GRA10* genomic target flank and a 967-bp 3’ *GRA10* genomic target flank to delete nucleotide 6215048 to 6217010 of the *GRA10* locus on chromosome VIII annotated as TGGT1_268900.

### Cell and Parasite Cultures

All parasites cultures were maintained *in vitro* by serial passages in human foreskin fibroblast (HFF) monolayers (ATCC SCRC-1041.1) in Eagle’s modified essential medium (EMEM) supplemented with 1% fetal bovine serum (FBS) at 36°C [51]. As specified in the text, certain experiments were performed using Dulbelcco’s modified Eagle’s medium (DMEM) supplemented with 10% FBS and 1% sodium pyruvate (D10 medium) or using lipid-free D10 medium (lfD10).

### Gene Replacements and Deletions

All strains used or developed in this study are listed in Table 1. Parasites were transformed following previously described methods [47]. Gene replacements using the *HXGPRT* marker were selected in 25 μg/mL mycophenolic acid (MPA) and 50 μg/mL xanthine (MPA+X). *HXGPRT* deletion was selected using 200 μg/mL 6-thioxanthine (6TX). Genotype verification of precisely targeted gene replacement and deletion events was performed by PCR as previously described [47, 48].

**Table 1:** Strains used or developed in this study.

Strain	Parent Strain	Genotype
RHΔ*ku80*Δ*hxgprt [48]*	RHΔ*ku80::HXGPRT [48]*	Δ*ku80*Δ*hxgprt*
RHΔ*ku80*Δ*gra2*::*HXGPRT*	RHΔ*ku80*Δ*hxgprt*	Δ*ku80*Δ*gra2*::*HXGPRT*
RHΔ*ku80*Δ*gra3*::*HXGPRT*	RHΔ*ku80*Δ*hxgprt*	Δ*ku80*Δ*gra3*::*HXGPRT*
RHΔ*ku80*Δ*gra4*::*HXGPRT*	RHΔ*ku80*Δ*hxgprt*	Δ*ku80*Δ*gra4*::*HXGPRT*
RHΔ*ku80*Δ*gra5*::*HXGPRT*	RHΔ*ku80*Δ*hxgprt*	Δ*ku80*Δ*gra5*::*HXGPRT*
RHΔ*ku80*Δ*gra6*::*HXGPRT*	RHΔ*ku80*Δ*hxgprt*	Δ*ku80*Δ*gra6*::*HXGPRT*
RHΔ*ku80*Δ*gra7*::*HXGPRT*	RHΔ*ku80*Δ*hxgprt*	Δ*ku80*Δ*gra7*::*HXGPRT*
RHΔ*ku80*Δ*gra8*::*HXGPRT*	RHΔ*ku80*Δ*hxgprt*	Δ*ku80*Δ*gra8*::*HXGPRT*
RHΔ*ku80*Δ*gra9*::*HXGPRT*	RHΔ*ku80*Δ*hxgprt*	Δ*ku80*Δ*gra9*::*HXGPRT*
RHΔ*ku80*Δ*gra2*Δ*hxgprt*	RHΔ*ku80*Δ*gra2*::*HXGPRT*	Δ*ku80*Δ*gra2*Δ*hxgprt*
RHΔ*ku80*Δ*gra3*Δ*hxgprt*	RHΔ*ku80*Δ*gra3*::*HXGPRT*	Δ*ku80*Δ*gra3*Δ*hxgprt*
RHΔ*ku80*Δ*gra4*Δ*hxgprt*	RHΔ*ku80*Δ*gra4*::*HXGPRT*	Δ*ku80*Δ*gra4*Δ*hxgprt*
RHΔ*ku80*Δ*gra2*Δ*gra4*::*HXGPRT*	RHΔ*ku80*Δ*gra2*Δ*hxgprt*	Δ*ku80*Δ*gra2*Δ*gra4*::*HXGPRT*
RHΔ*ku80*Δ*gra2*Δ*gra6*::*HXGPRT*	RHΔ*ku80*Δ*gra2*Δ*hxgprt*	Δ*ku80*Δ*gra2*Δ*gra6*::*HXGPRT*
RHΔ*ku80*Δ*gra4*Δ*gra6*::*HXGPRT*	RHΔ*ku80*Δ*gra4*Δ*hxgprt*	Δ*ku80*Δ*gra4*Δ*gra6*::*HXGPRT*
RHΔ*ku80*Δ*gra3*Δ*gra5*::*HXGPRT*	RHΔ*ku80*Δ*gra3*Δ*hxgprt*	Δ*ku80*Δ*gra3*Δ*gra5*::*HXGPRT*
RHΔ*ku80*Δ*gra3*Δ*gra7*::*HXGPRT*	RHΔ*ku80*Δ*gra3*Δ*hxgprt*	Δ*ku80*Δ*gra3*Δ*gra7*::*HXGPRT*

### Western Blots

Parasites were isolated from freshly lysed cultures and resuspended in Laemmli buffer. Proteins were separated by 13% SDS-PAGE (non-reduced conditions), transferred to nitrocellulose membranes and detected using the following primary antibodies: mAb TG17.179 anti-GRA2 (1:15,000) [16], mAb T6.2H11 anti-GRA3 (1:10,000) [52], rabbit anti-GRA4 (1:10,000) [53], mAb TG17.113 anti-GRA5 (1:5,000) [16], rabbit anti-GRA6 (1:20,000) [53], mAb BATO 214 anti-GRA7 (1:15,000) [54], mAb 3.2 anti-GRA8 (1:10,000) [55], rabbit anti-GRA9 (1:2,500) [56], rabbit anti-actin (1:10,000) [57] or mAb TG054 anti-SAG1 (1:15,000) [58] (antibodies purchased from the Biotem company, Apprieu, France or kindly provided by L. D. Sibley, Washington University School of Medicine, Saint-Louis, MO; D. Jacobs, Innogenetics-Fujirebio Europe N.V., Ghent, Belgium; G.E. Ward, University of Vermont College of Medicine, Burlington, VT; W. Daübener, Heinrich Heine Universität, Düsseldorf, Germany). Proteins were detected with horseradish peroxidase (HRP)-conjugated secondary antibodies (1:20,000; Jackson Immunoresearch Laboratories) and the peroxidase activity was visualized by chemiluminescence using the Supersignal ECL system (Pierce Chemical).

### Indirect immunofluorescence

Confluent HFFs were grown on glass coverslips and infected overnight with parasites. For experiments using lipid free medium, HFFs were equilibrated in lfD10 and parasites were passed three times in lfD10 prior to infection experiments. Infected cells were fixed in 5% formaldehyde, permeabilized with 0.002% saponin and blocked in 5% goat serum-5% FBS (in Phosphate Buffered Saline (PBS)). Cells were then incubated with primary antibodies for 1 hr in 1% FBS, 0.002% saponin. Primary antibodies used in this study included mAbTG17.179 anti-GRA2 (1:500), mAb T6.2H11 anti-GRA3 (1:500), rabbit anti-GRA4 (1:500), rabbit anti-GRA6 (1:500), mAb BATO 214 anti-GRA7 (1:500), mAb 3.2 anti-GRA8 (1:500), rabbit anti-GRA9 (1:500), and mAb TG 05.54 anti-SAG1 (1: 500). Infected cells were washed and incubated for 1 h with the following secondary antibodies: goat anti-mouse IgG (H+L)-Alexa 488 (1:500, Jackson) or goat anti-mouse IgG (H+L) Alexa 594 (1:500, Jackson) or goat anti-rabbit IgG (H+L) Alexa 488 (1:500, Jackson). Coverslips were then incubated with 5 μg/mL Hoechst 33342 for 10 min to stain nuclei, mounted in Mowiol and observed using a 100X objective on an Axioplan II microscope (Zeiss). Images were acquired using a Zeiss black and white camera and the Axio Vision software (release 4.7.1).

### Transmission Electron Microscopy (TEM)

Monolayers of HFFs were grown to confluence on Permanox slides, infected for 24 h in D10- or in lfD10 medium, rinsed with PBS, fixed for 2 h with glutaraldehyde diluted in 0.2 M NaPO_4_ pH 7.4 and processed for TEM, as previously described [25]. To allow preservation of the IVN, the infected cells were flat embedded and sectioned en face.

### Intracellular Replication and Infectivity Assays

The intracellular growth rate was determined in HFFs in a 30-hr growth assay. Freshly lysed parasites were filtered through a Nuclepore filter to remove host cell debris and used to infect HFFs at a multiplicity of infection (MOI) of ~ 0.1. An hour after infection the monolayer was washed with PBS and fresh medium was added to the culture. Cultures were prepared in duplicate. At 30 h post-infection the number of parasites/vacuole was scored from 250 PVs per parasite strain to determine the average number of parasites per vacuole in an HFF cell with a single PV. Samples were represented as the average number of parasite(s) per PV (± SEM). Replication rates were compared between parental and knockout strains using a Student’s *t*-test, with significance being represented as a *P* value <0.05.

For high throughput replication and infectivity assays, HFF cells were plated in 96 well plates (10,000 cells per well). Cells were grown for 48 h, rinsed with PBS and 40,000 parasites of each strain (MOI ~1–2) were deposited into the wells (250 μL per well). Plates were spun at 400 rpm for 1 min, then incubated for 2 h to allow parasite invasion. After 3 washes in PBS, fresh D10 medium was added to each well and the plates were returned to culture for 24 h. The infected monolayers were washed with PBS, incubated with 5 μg/mL Hoechst 33342 for 10 min, rinsed, saturated and permeabilized for 15 min with 5% goat serum– 0.1% triton-X100 (PBS-T), and incubated for 45 min with mAb TG17.043 anti-GRA1 (1:500, Biotem) in PBS-T. After washes, cells were incubated with goat anti-mouse IgG-Alexa 488 (1:500, Jackson), washed again, and analyzed by high content screening microscopy using an Olympus IX8 inverted microscope equipped with a black and white Orca ER Camera, and a LUCPLLN 20xPH1 objective. Images of the entire surface of each well were analyzed using the ScanR software. The percentage of infected cells, the total number of parasites per PV as well as the percentage of PVs containing 1, 2, 4, 8, 16 parasites was determined for >5,000 PVs per strain. Statistical analyses were performed using a Student’s *t*-test, with significance being represented as a *P*-value <0.05.

### Ethics Statement

All animal experiments were performed in strict accordance with the U.S.A. Public Health Service Policy on Humane Care and Use of Laboratory Animals. Animals experiments were conducted in an AAALAC approved facility. All animal protocols (protocol bzik.dj2) were approved by the Institutional Animal Care and Use Committee (Dartmouth College: Animal Welfare Assurance Number #A3259-01). The humane endpoint of weight loss was used to determine when animals were euthanized (≥ 17% body weight loss). Animals were monitored daily until weight loss occurred then animals were monitored at least twice a day. If the humane endpoint was reached, animals were euthanized using CO_2_ and additionally by cervical dislocation. All efforts, including providing diet gel recovery to mice with weight loss, were made to minimize suffering.

### Acute infection

Adult female CD1 mice were obtained from Charles River Laboratory and mice were maintained in Techniplast Seal Safe mouse cages on vent racks and provided with enrichment materials at the Dartmouth-Hitchcock Medical Center (Lebanon, NH) mouse facility. Acute virulence was determined by a single intraperitoneal injection of the indicated numbers of tachyzoites into groups of four to eight week old female CD-1 mice (17-21g) per experiment. Studies were done in a blinded manner to minimize subjective bias. No unexpected deaths occurred during experiments. Survival was monitored for 30 days and the percentage of surviving animals was determined by the number of animals that survived / the total number of animals that were infected x 100. In all experiments, plaque forming units (PFUs) to tachyzoite ratios were determined at the time of parasite inoculation to verify infectivity of parasite preparations. Survival was analyzed by the Kaplan-Meier method and curves were compared using the log rank (Mantel-Cox) test in GraphPad Prism.

## Results

### Targeted deletion of GRA2, GRA3, GRA4, GRA5, GRA6, GRA7, GRA8, and GRA9 gene loci

**Fig 1:**
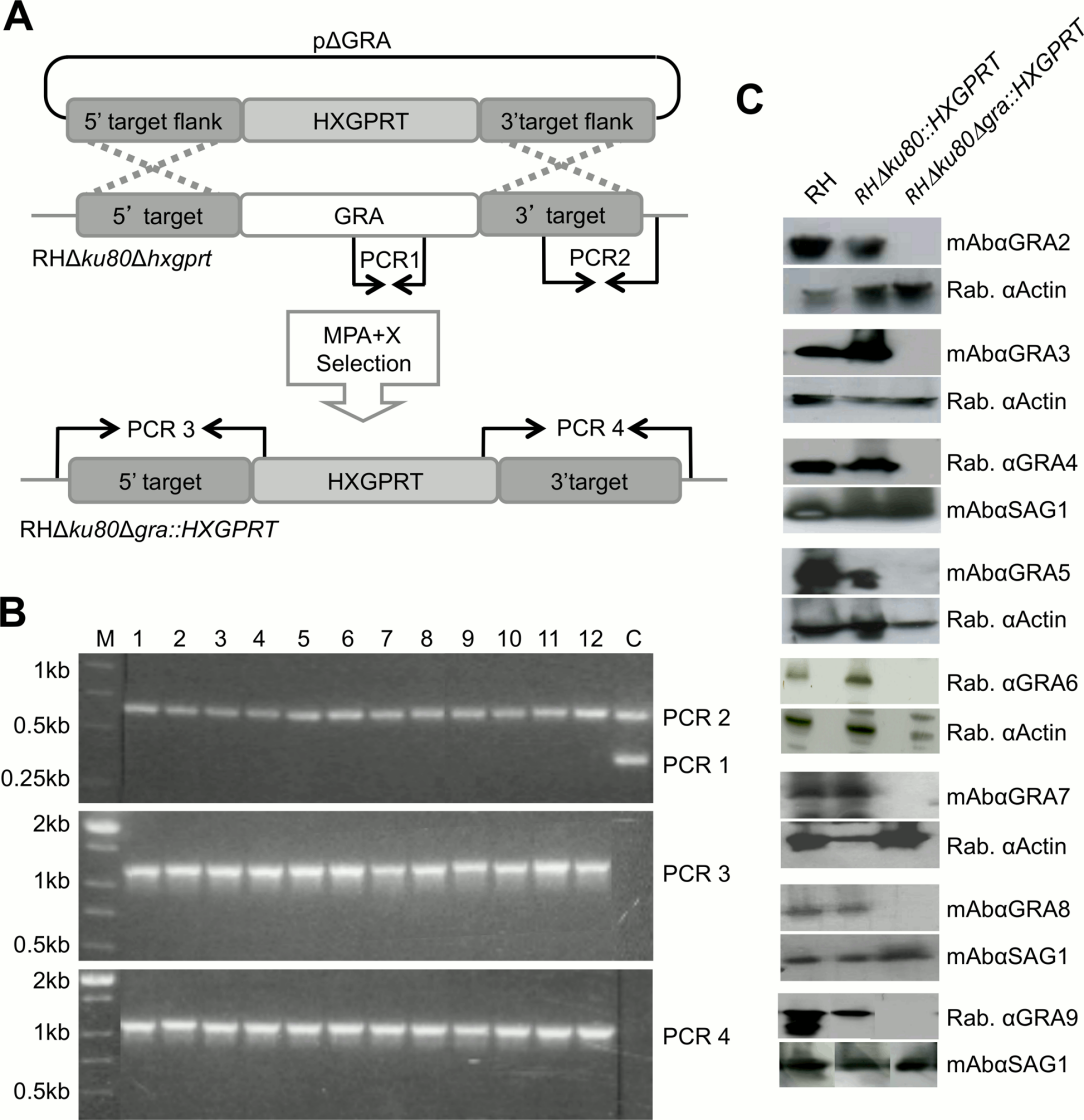
Construction and validation of the single *GRA2-9* knockout strains. A) Strategy for disruption of a *GRA* gene locus by double crossover homologous recombination in type I RHΔ*ku80*Δ*hxgprt* using a plasmid, pΔGRA, engineered to contain the 5’ and 3’ target flanks surrounding the *HXGPRT* selectable marker. Successful recombination events were selected by mycophenolic acid plus xanthine (MPA+X). This strategy is representative of each of the gene knockout attempts. B) Validation of the Δ*gra4* targeted gene deletion by PCR. The control parental strain is positive for PCR 1 (~ 380 bp) and PCR 2 (~ 670 bp) but negative for PCR 3 (~ 1,100 bp) and PCR4 (~ 1,200 bp). Clones 1–12 exhibit the banding pattern of a targeted Δ*gra4* gene knockout,. M: DNA size ladder. C) Western blot validation of the deletion of GRA2-9. Cell lysates from the equivalent of 2 x 10^7^ parasites were loaded per lane, separated on a 13% SDS-PAGE (non-reduced conditions), probed with primary antibodies (indicated on the side of the gels), then with goat anti-mouse IgG or goat anti-rabbit IgG, both coupled to peroxidase and visualized by chemiluminescence.

To further investigate the role of the first 10 historically identified GRA proteins (*GRA1-10*), we precisely targeted knockouts at each of these unique *GRA* gene loci using the virulent type I genetic background deficient in nonhomologous end-joining [48]. Gene knockout targeting plasmids were constructed according to the representative pΔGRA targeting plasmid shown in Fig 1A. These targeting plasmids position the *HXGPRT* selectable marker between the 5’ and 3’ DNA flanks of the *GRA* gene of interest, thereby replacing the GRA protein coding sequence with the *HXGPRT* selectable marker [59].

**Fig 2:**
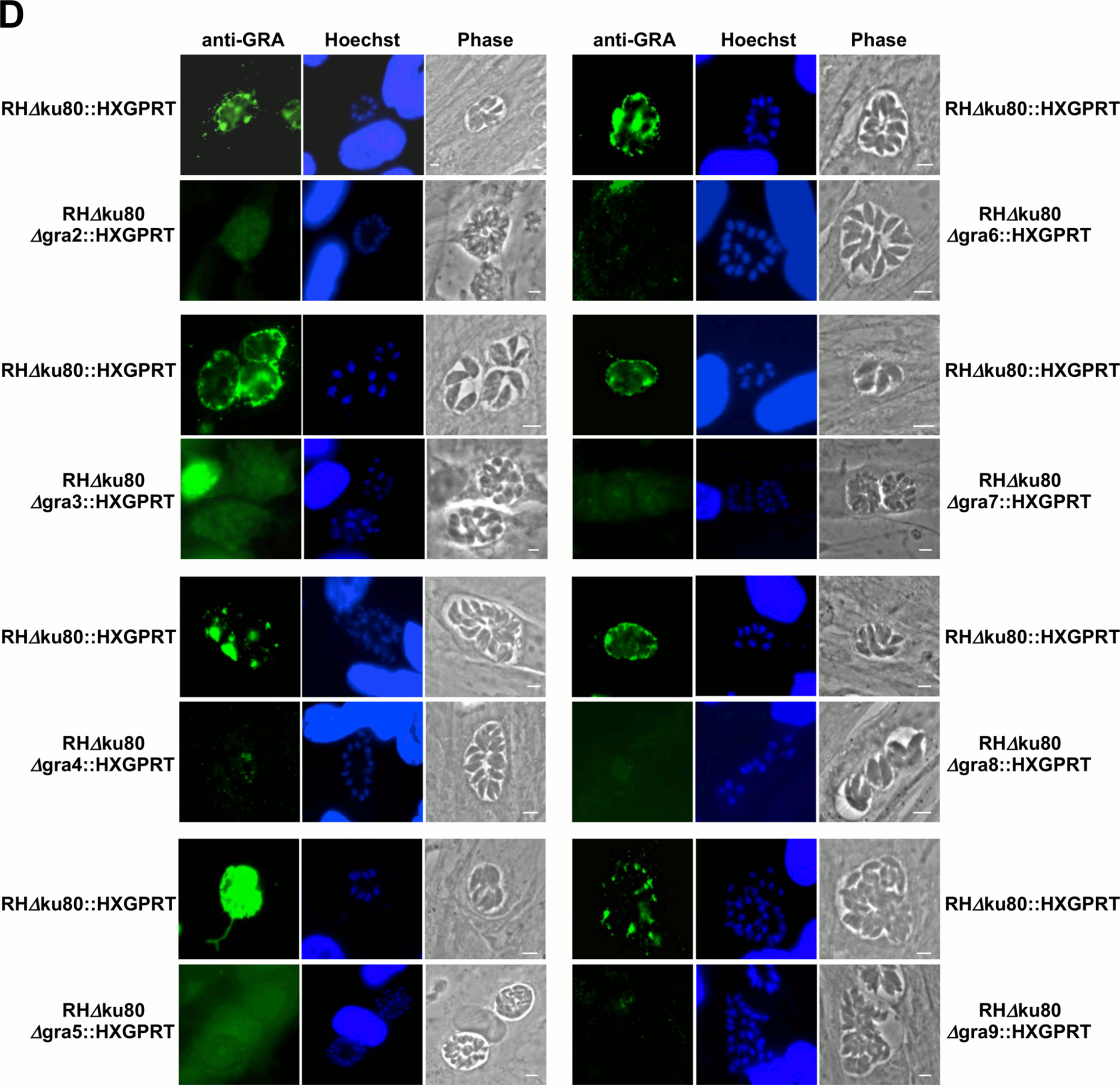
Indirect immunofluorescence assay verifies deletion of GRA proteins. IFA validation of the deletion of GRA2-9. HFFs were infected overnight with Δ*gra* knockout strains or with the parental strain. Infected cells were fixed, permeabilized with 0.002% saponin and incubated with the appropriate primary antibodies (see Material and Methods), followed by Alexa 488-coupled goat anti-mouse IgG or goat anti-rabbit IgG secondary antibodies. After labeling both the host- and parasite nuclei were revealed with Hoechst 33342; coverslips were mounted in Mowiol and observed by epifluorescence. To distinguish the shape of ∆gra mutants’ vacuoles in the absence of fluorescent signal, we chose to artificially increase their exposure time, which may lead to some artificial background noise. Scales: 5 μm.

Following transfection into the RHΔ*ku80*Δ*hxgprt* parental strain [48] and MPA+X selection, we isolated knockout strains deleted for *GRA2* (Δ*gra2*), *GRA3* (Δ*gra3*), *GRA4* (Δ*gra4*), *GRA5* (Δ*gra5*), *GRA6* (Δ*gra6*), *GRA7* (Δ*gra7*), *GRA8* (Δ*gra8*), and *GRA9* (Δ*gra9*). Knockouts at the *GRA1* and *GRA10* loci were not obtained despite repeated transfection-selection attempts. The genotypes of the Δ*gra2*-*9* knockout strains (Table 1) were validated by PCR as shown in Fig 1B for the Δ*gra4* strain. The deletion of each GRA protein was also confirmed by western blot (Fig 1C), and also by indirect immunofluorescence in HFFs infected overnight (Fig 2).

### The loss of single GRA genes does not affect the replication rate

GRA proteins have been proposed to facilitate interactions with the host cell and to potentially mediate nutrient acquisition [60]. We used two different methodologies to investigate whether GRA2-9 are necessary for parasite growth *in vitro*. First, we infected HFFs with each of the knockout strains and monitored the parasite replication rate by directly counting the number of parasites per PV in 250 PVs at 30 h post-infection by light microscopy. Using this direct method, we did not observe any significant defect in replication rates determined by scoring the number of parasites per PV (Table 2).

**Table 2:** Intracellular replication rate of single Δ*gra* knockout parasite strains.

Strain	Mean number of parasites per vacuole^a^ (±SEM)	*P* (compared to the control strain)^b^	
		Significance	Control strain genotype
RHΔ*ku80::HXGPRT [48]*	19.85 ± 0.76		
RHΔ*ku80*Δ*gra2*::*HXGPRT*	19.06 ± 1.29	NS	RHΔ*ku80*::*HXGPRT*
RHΔ*ku80*Δ*gra3*::*HXGPRT*	17.12 ± 1.81	NS	RHΔ*ku80*::*HXGPRT*
RHΔ*ku80*Δ*gra4*::*HXGPRT*	18.94 ± 1.63	NS	RHΔ*ku80*::*HXGPRT*
RHΔ*ku80*Δ*gra5*::*HXGPRT*	22.44 ± 0.83	NS	RHΔ*ku80*::*HXGPRT*
RHΔ*ku80*Δ*gra6*::*HXGPRT*	20.82 ± 0.12	NS	RHΔ*ku80*::*HXGPRT*
RHΔ*ku80*Δ*gra7*::*HXGPRT*	23.38 ± 0.34	NS	RHΔ*ku80*::*HXGPRT*
RHΔ*ku80*Δ*gra8*::*HXGPRT*	19.72 ± 2.23	NS	RHΔ*ku80*::*HXGPRT*
RHΔ*ku80*Δ*gra9*::*HXGPRT*	21.60 ± 1.78	NS	RHΔ*ku80*::*HXGPRT*

^a^ The number of parasites per vacuole was scored at 30 h PI in HFF cells containing a single PV.

^b^ *P* value was determined by a Student’s *t*-test. NS, not significant at *P* ≤ 0.05.

**Fig 3:**
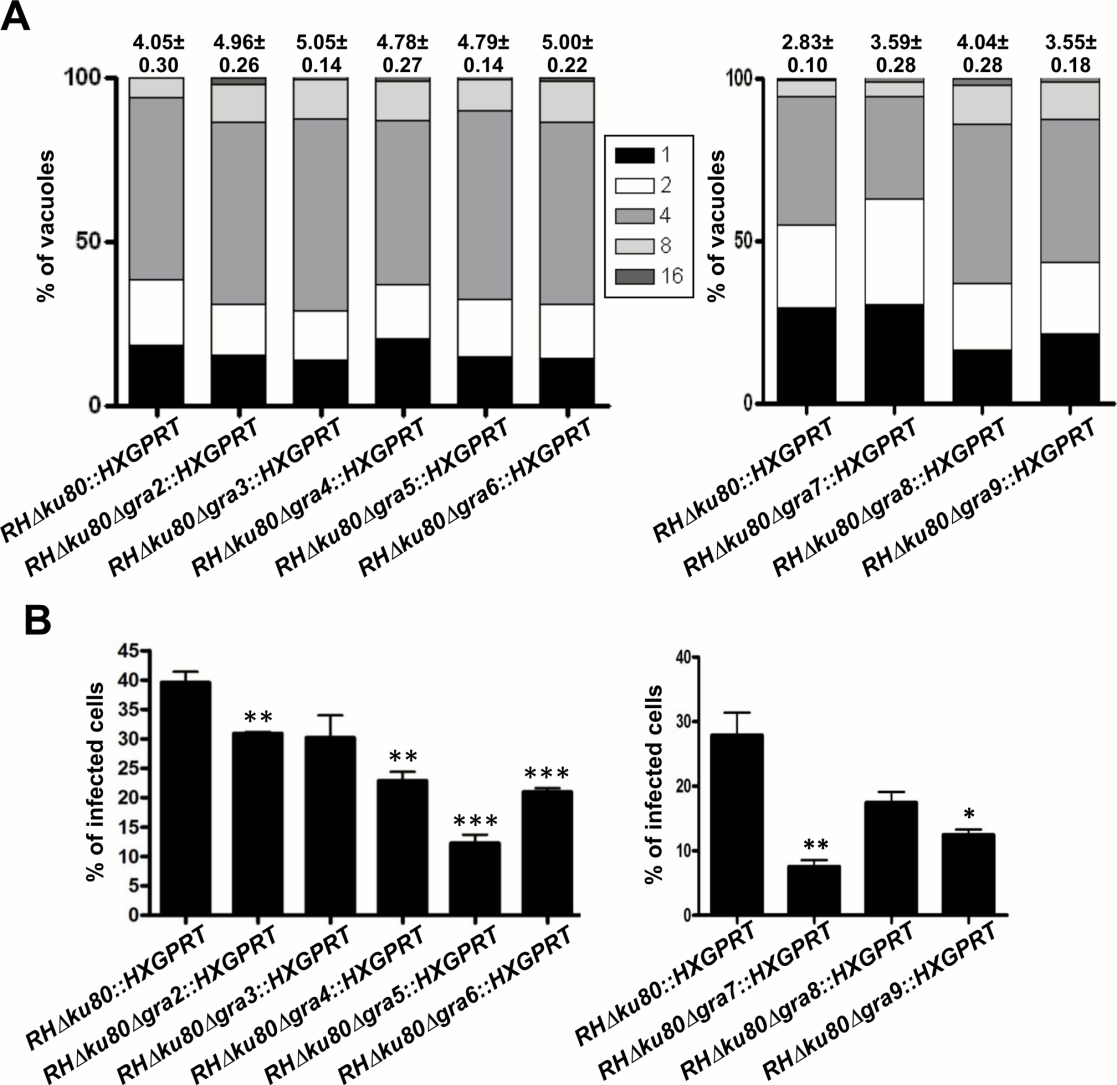
Replication and infection rates of single Δ*gra2-*9 gene knockout strains calculated by high throughput microscopy. A) Replication rate of each of the Δ*gra2-*9 strains compared to that of the parental strain. The percentage of PVs containing 1, 2, 4, 8 or 16 parasites per vacuole was calculated from three coverslips of infected HFFs. The numbers above each bar represent the average number of parasites per vacuole calculated from the total number obtained the three coverslips. The values are representative of one experiment out of three, which provided similar results. B) Percentage of HFFs infected by each Δ*gra2-9* knockout strain *versus* the parental Δ*ku80* strain. The infection rate was calculated from the number of PVs observed on three coverslips. Comparisons between the parental strain and each of the knockout strains were performed using a Student’s *t*-test and asterisks indicate significant differences. *: *p* = 0.0128 for Δ*gra9*; **: *p* = 0.0099 for Δ*gra2*, 0.0022 for Δ*gra4*, 0.0051 for Δ*gra7*; ***: *p* = 0.0003 for Δ*gra5* and 0.0007 for Δ*gra6*. The values are representative of one experiment out of three, which provided similar results. The total number of PVs observed in A and B are respectively: parental Δ*ku80* strain, 5,757 (left panel) and 5,206 (right panel); Δ*gra2*, 5,200; Δ*gra3*, 5,173; Δ*gra4*, 6,176; Δ*gra5*, 5,845; Δ*gra6*, 6,311; Δ*gra7*, 5,165; Δ*gra8*, 5,177; Δ*gra9*, 5,065.

To extend this analysis and potentially detect subtle growth defects by analyzing a larger sample size of more than 5,000 PVs, we used a high throughput automated immunofluorescence method to quantify the percentage of PVs containing 1, 2, 4, 8, 16 parasites and determine the average number of parasites per vacuole (replication rate), as well as the number of infected HFF cells (infection rate). The results confirmed the data shown in Table 2 revealing there was no significant reduction in the replication rate of single GRA knockout strains (Fig 3A), though the Δ*gra7* knockout did exhibit a slight increase in the percentage of PVs containing only 1 or 2 parasites. In contrast, a significant decrease in the infection rate was observed for the Δ*gra2*, Δ*gra4*, Δ*gra5*, Δ*gra6*, Δ*gra7*, and Δ*gra9* knockout strains (Fig 3B).

### Alteration of the intravacuolar network after deletion of GRA2, GRA6 and GRA7

**Fig 4:**
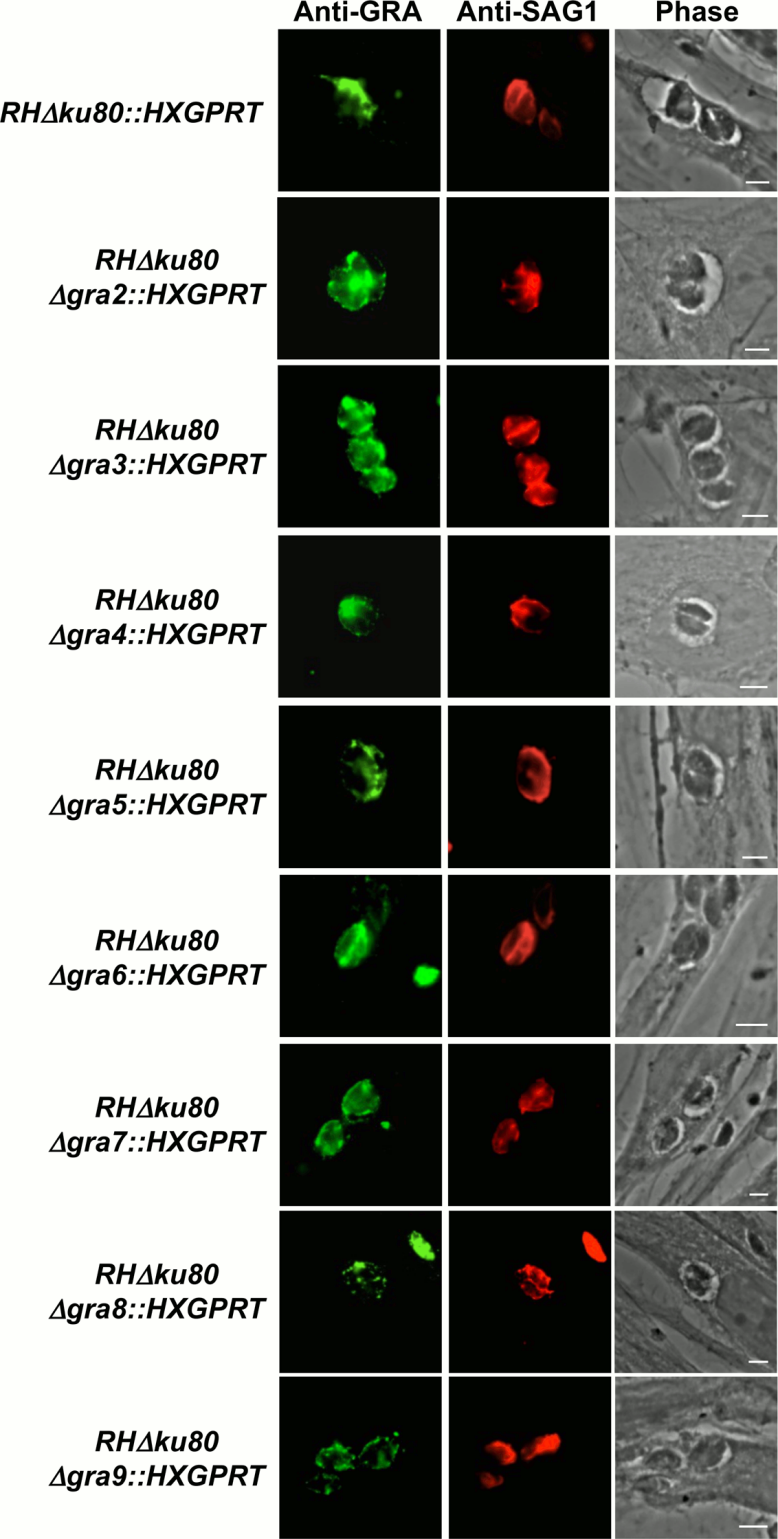
PV formation by Δ*gra2-9* single knockout strains in normal and lipid-free media. HFFs were pre-equilibrated in lipid-free D10 media (lfD10), then infected overnight with a single Δ*gra* knockout parasite strain or the Δ*ku80* parental strain. All parasite strains were passaged 3 times in lfD10 medium prior to infecting HFFs. Infected cells were fixed, permeabilized with 0.002% saponin, incubated with rabbit serum anti-GRA6 (all strains but Δ*gra6*) or with rabbit serum anti-GRA4 (Δ*gra6*) and with mAb anti-SAG1, visualized with goat anti-rabbit IgG coupled to Alexa 488 and goat anti-mouse IgG coupled to Alexa 594, mounted in Mowiol and observed by epifluorescence. To distinguish the shape of ∆gra mutants’ vacuoles in the absence of fluorescent signal, we chose to artificially increase their exposure time, which may lead to some background noise. Scales: 5 μm.

To test whether the absence of lipids in growth medium would reveal any distinct morphologic changes in the Δ*gra2-9* knockout strains, we infected HFFs in normal D10 medium or HFFs that were pre-equilibrated in lfD10 medium. Each of the *GRA* knockout strains developed normal appearing PVs in both D10 (Fig 2) and in lfD10 media as assessed by indirect immunofluorescence assays (Fig 4).

**Fig 5:**
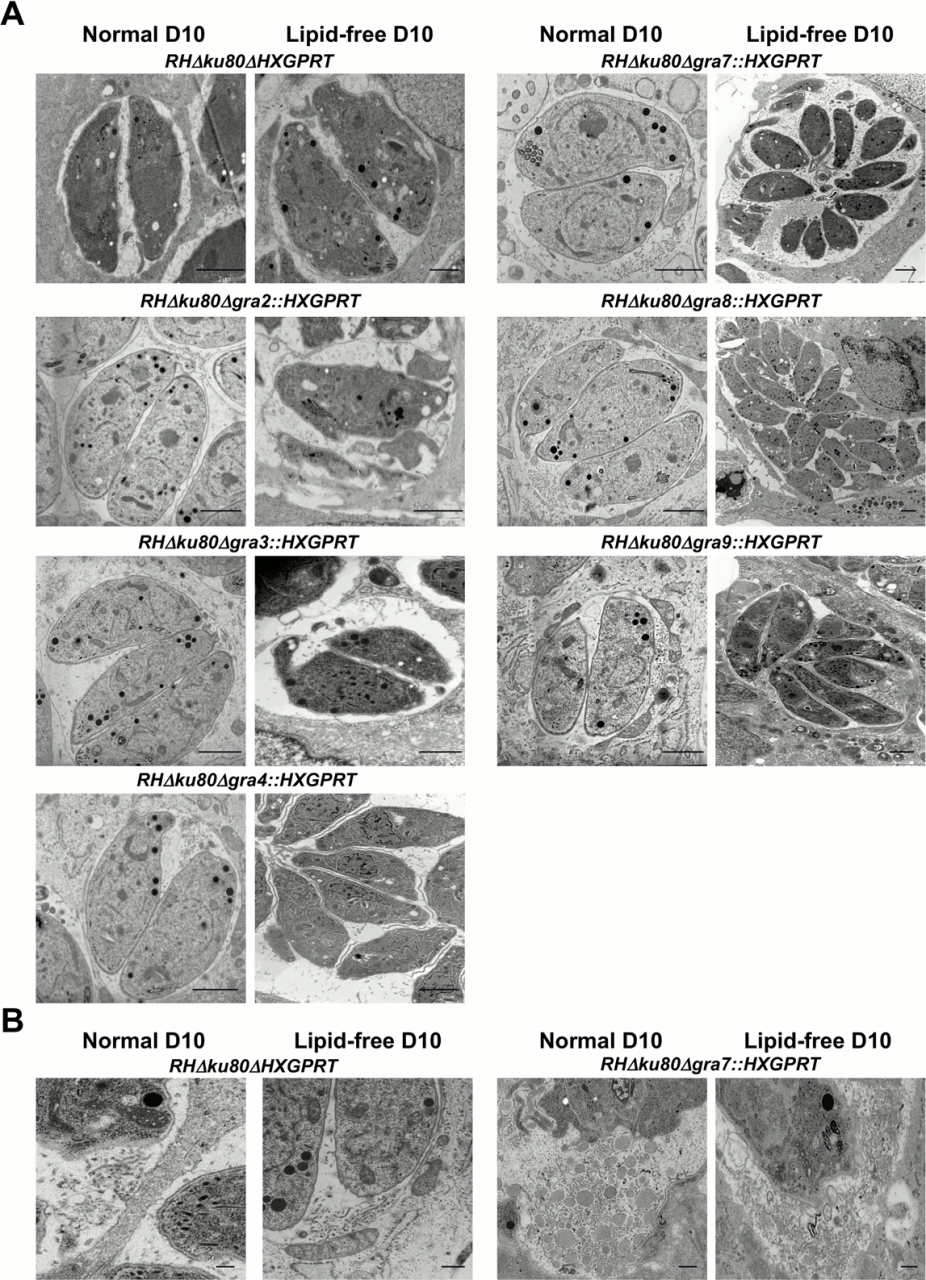
Visualization of the Δ*gra2-9* knockout parasites and PV morphology by transmission electron microscopy. A) HFFs and parasites were cultured in D10 medium (“Normal D10”) or pre-equilibrated in lipid-free D10 medium (“Lipid-free D10”). Host cells were infected overnight with Δ*gra2-9* knockout or parental strains, fixed, and then processed for transmission electron microscopy. Scale: 2 μm. B) Magnification of the posterior end of an intracellular Δ*gra7* knockout parasite in “Normal D10” and in “Lipid-free D10” showing the hyper-formation of the IVN. Scales: 2 μm.

Therefore, we assessed the morphology of the parental and *GRA* knockout strains cultured *in vitro* in D10 or lfD10 media by transmission electron microscopy (TEM). In the Δ*gra2* (Fig 5A) and Δ*gra6* (data not shown) knockout strains the parasites, their dense granules and PVM appeared normal; however, no IVN was observed in mature PVs grown in normal D10 medium (Fig 5A and data not shown), confirming previously reported phenotypes [25]. The absence of IVN in the mature PV of Δ*gra2* and Δ*gra6* strains was also observed in lfD10 medium (Fig 5A and data not shown). Interestingly, the Δ*gra7* knockout exhibited an increase in the density of membranous tubules and vesicles that form the IVN of parasites grown both in D10 and lfD10 media (Fig 5A and 5B). No major morphologic changes in the parasites or their PV were observed in other *GRA* knockout strains cultured in normal D10 or delipidated lfD10 conditions (Fig 5 and data not shown).

### Targeted double deletions of dense granule genes

**Fig 6:**
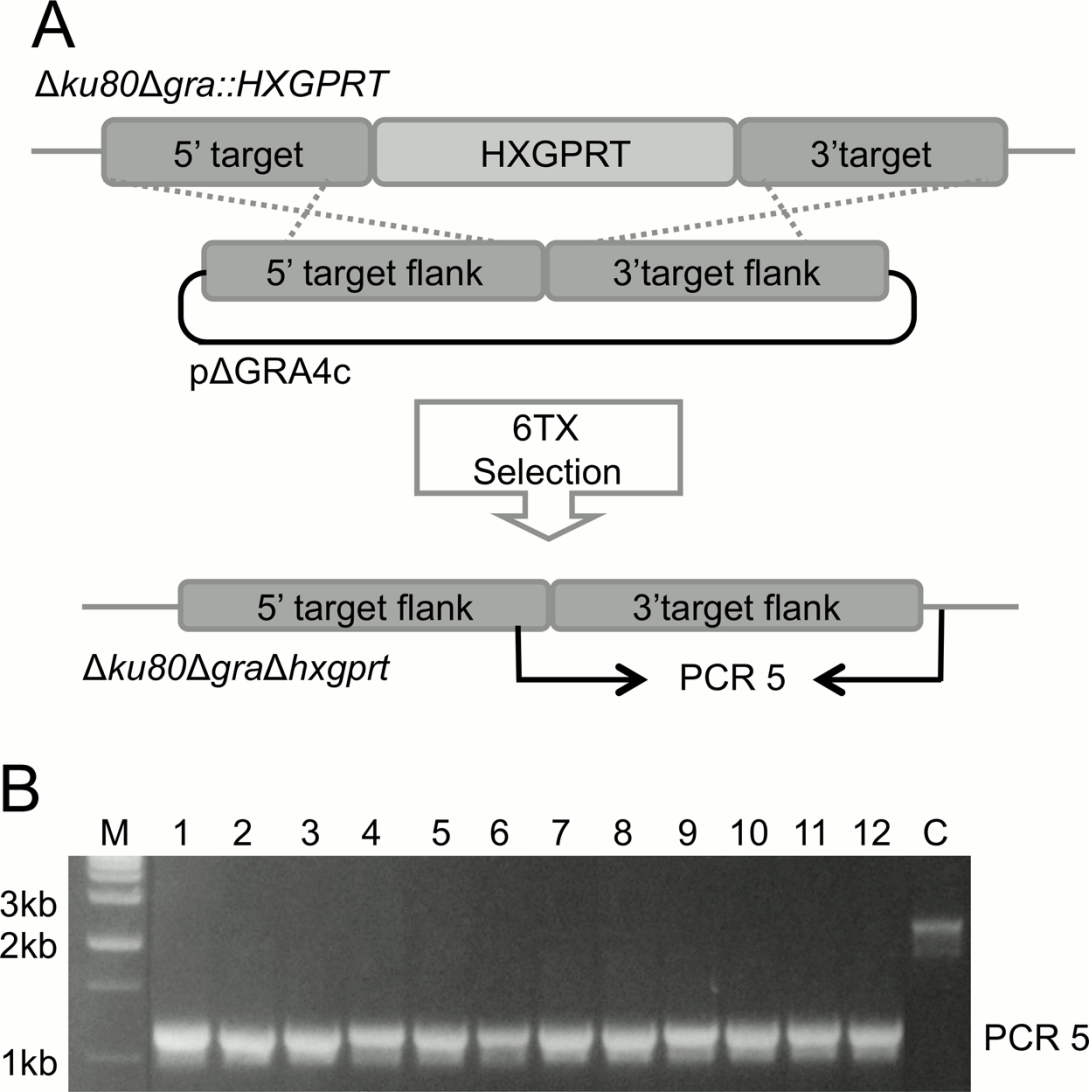
Strategy for removal of the *HXGPRT* selectable marker from a *GRA* gene locus. A) Removal of the *HXGPRT* selectable marker from RHΔ*ku80*Δ*gra*::*HXGPRT*. This strategy is representative of each of the *HXGPRT* removal attempts. Following transfection with an *HXGPRT*-excised plasmid, clones lacking *HXGPRT* but containing the 5’ and 3’ flanking regions of the original *GRA* locus were generated by double-crossover homologous recombination, using 6TX selection. B) Validation of the *HXGPRT* removal from the *gra4* gene locus. The parental strain exhibits a PCR5 amplicon of ~ 2,300 bp, corresponding to a DNA fragment that includes the *HXGPRT* coding sequence. Clones 1–12 exhibit a PCR5 amplicon of ~ 1,100 bp, which corresponds to the removal of *HXGPRT*. M: DNA size ladder; C: control parental strain.

We generated several double *GRA* knockout strains for GRA proteins that were previously reported to associate or co-localize [61] to assess whether these GRA proteins may provide redundant functions. The *HXGPRT* marker present in the disrupted *GRA* locus was excised using the pΔGRAc plasmid and selection in 6-thioxanthine to generate the corresponding Δ*gra*Δ*hxgprt* strain (Fig 6A), and genotypes were validated by PCR (Fig 6B). The Δ*gra*Δ*hxgprt* knockout strains were then used to generate double *GRA* knockout strains following the forward strategy illustrated in Fig 1A using MPA + X selection. Five double *GRA* knockout mutants were generated: Δ*gra2*Δ*gra4*, Δ*gra2*Δ*gra6*, Δ*gra4*Δ*gra6*, Δ*gra3*Δ*gra5* and Δ*gra3*Δ*gra7* (Table 1). Interestingly, despite several attempts, we failed to isolate a Δ*gra2*Δ*gra4*Δ*gra6* triple knockout strain even though each of the three corresponding double *GRA* knockout gene combinations was successfully isolated (Table 1).

### Double GRA knockout strains display defects in replication rate

We measured the *in vitro* replication rates of the double *GRA* knockout strains via direct counting of parasites in > 250 PVs 30 h post-infection. The Δ*gra4*Δ*gra6*, Δ*gra3*Δ*gra5* and Δ*gra3*Δ*gra7* knockout strains exhibited mild defects in their *in vitro* replication rate (Table 3). No growth replication defects were observed for the Δ*gra2*Δ*gra4* or Δ*gra2*Δ*gra6* knockout strains (Table 3).

**Table 3:** Intracellular replication rate of double Δ*gra* knockout parasite strains.

Strain	Mean number of parasites per vacuole^a^ (±SEM)	*P* (compared to the control strain)^b^	
		Significance	Control strain genotype
RHΔ*ku80*::*HXGPRT (48)*	19.85 ± 0.76		
RHΔ*ku80*Δ*gra2*Δ*gra4*::*HXGPRT*	16.89 ± 1.12	NS	RHΔ*ku80*::*HXGPRT*
RHΔ*ku80*Δ*gra2*Δ*gra6*::*HXGPRT*	18.97 ± 0.56	NS	RHΔ*ku80*::*HXGPRT*
RHΔ*ku80*Δ*gra4*Δ*gra6*::*HXGPRT*	15.53 ± 0.68	S	RHΔ*ku80*::*HXGPRT*
RHΔ*ku80*Δ*gra3*Δ*gra5*::*HXGPRT*	14.14 ± 0.75	S	RHΔ*ku80*::*HXGPRT*
RHΔ*ku80*Δ*gra3*Δ*gra7*::*HXGPRT*	14.85 ± 0.60	S	RHΔ*ku80*::*HXGPRT*

^a^ The number of parasites per vacuole was scored at 30 h post-infection in HFF cells containing a single PV.

^b^ *P* value was determined by a Student’s T-test. S, significant at *P* ≤ 0.05; NS, not significant.

### Deletion of GRA2-9 does not affect acute infection or virulence in vivo

**Fig 7:**
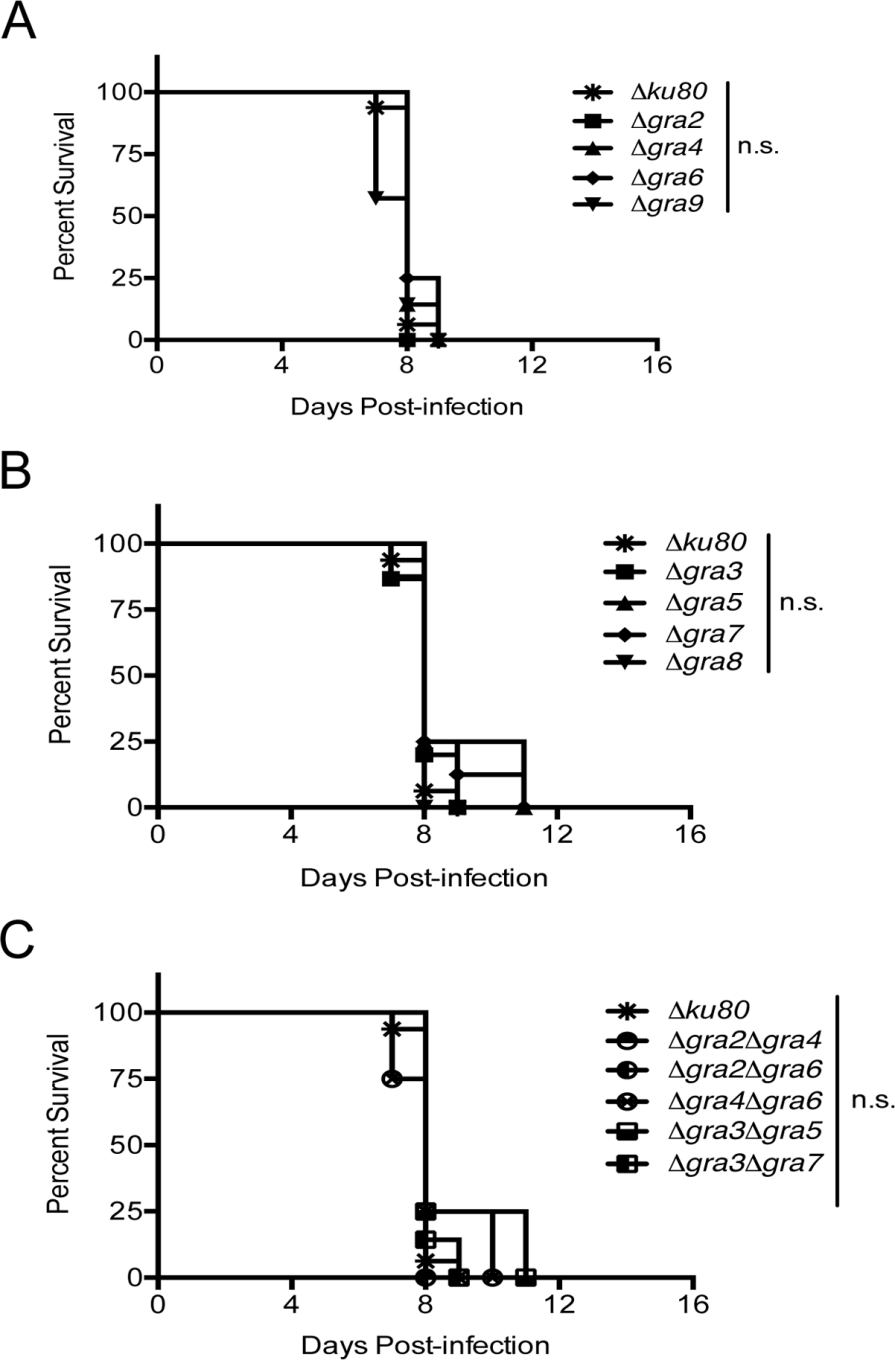
Virulence of single and double *GRA* knockout strains in CD1 mice. One hundred tachyzoites of the parental (Δ*ku80*) or knockout (Δ*gra)* strains were injected intraperitoneally (i.p.) into 8 week old female CD1 mice and survival was followed for 30 days (n = 8 for Δ*gra3*, Δ*gra4*, Δ*gra7*, Δ*gra9*, Δ*gra3*Δ*gra5* and Δ*gra3*Δ*gra7*, n = 4 for Δ*gra2*, Δ*gra5*, Δ*gra6*, Δ*gra8*, Δ*gra2*Δ*gra4*, Δ*gra2*Δ*gra6*, and Δ*gra4*Δ*gra6*). A) Survival of mice infected with parasites containing single *GRA* knockouts for IVN-localized GRA proteins. B) Survival of mice infected with parasites containing single *GRA* knockouts for PVM-localized GRA proteins. C) Survival of mice infected with parasites containing double *GRA* knockouts. Significance was determined by a Student’s t-test comparing the knockout strain with the parental.

Previous work identified several GRA proteins (GRA2, GRA6, and GRA7) as playing a role in type I parasite virulence during acute infection [25, 31, 37, 42]. To examine the virulence of the Δ*gra2-*9 knockout strains, we infected CD-1 female mice intraperitoneally with 100 tachyzoites (parental RH strain LD_100_ = 1 parasite) and monitored their survival. Absence of these individual GRA proteins (Fig 7A and 7B) or multiple GRA proteins (Fig 7C) did not increase or decrease virulence during acute infection. All mice succumbed to the infection within 8–12 days.

## Discussion

The aim of this study was to apply a more systematic and reliable genetic approach using the virulent type I strain Δ*ku80* genetic background to examine or re-examine phenotypes associated with the absence of the dense granule proteins GRA1-10. Previously, only one of these ten GRA proteins (GRA7, [31]) was knocked out in a background that eliminates off-target mutations that could influence phenotypes in non-Δ*ku80* strains [47]. Using the virulent type I Δ*ku80* genetic model that enables efficient and precise targeted deletion [46–48], we successfully generated parasite strains containing single and double deletions of the *GRA* loci *GRA2-9* (Table 1). This study is the first to report results on knockouts at the *GRA3*, *GRA4*, *GRA8* and *GRA9* loci, as well as results on the double *GRA* knockouts Δ*gra2*Δ*gra4*, Δ*gra4*Δ*gra6*, *gra3*Δ*gra5* and Δ*gra3*Δ*gra7* in the virulent type I RH genetic background.

While knockouts were readily isolated at the *GRA2-9* loci, we could not isolate a Δ*gra1* or a Δ*gra10* knockout, suggesting the possibility that these *GRA* genes are required for parasite growth or survival, though additional experiments would be necessary to validate this conclusion. Previously, RNA-knockdown of *GRA10* expression severely inhibited growth of the parasite *in vitro* [62]. *GRA1* encodes a GRA protein that localizes to the PV lumen as a calcium-binding [63], soluble protein [13]. This unique localization and the inability to knockout these genes in a type I strain suggests that GRA1 and GRA10 may participate in essential growth functions. The potential essentiality of the *GRA1* and *GRA10* genes could be further addressed in the future using either an inducible type I deletion scheme such as Cre-recombinase [64], an inducible type I expression scheme [65], the CRISPR (clustered regularly interspaced short palindromic repeats)/CAS9 technology [66], or alternatively to apply the recently developed type II Δ*ku80* genetic model [46] which exhibits extremely rare or undetectable off-site targeting in comparison to type I Δ*ku80* genetic models [47].

Each of the GRA2-9 proteins contains either a transmembrane hydrophobic alpha-helix or amphipathic alpha-helices [67] that allow for insertion into and localization at the PVM and/or the IVN membranes. In addition, GRA2 and GRA6 induce formation and maturation of the IVN [25, 26] GRA7 was previously identified as a GRA protein possessing the ability to tubulate membranes *in vitro* [29]. We examined the parasite and PV morphologies of single *GRA* knockout strains cultured in both normal D10- and lipid free lfD10 media by indirect immunofluorescence and TEM. Interestingly, any phenotype observed in the D10 medium was also present in the delipidated lfD10 medium. For all single *GRA* knockout strains the parasites, their dense granules and their PVM appeared normal in D10 as well as in lfD10 medium. Any morphological differences in the knockouts occurred only in the IVN structures, reinforcing the importance of certain GRA proteins as key regulators of the formation and maintenance of the IVN. In corroboration with previous reports [25], the Δ*gra2* and the Δ*gra6* knockout strains lacked an IVN in D10 and in delipidated lfD10 media. In contrast, the Δ*gra7* knockout strain exhibited a hyper-formation of the IVN in both D10 and lfD10 media. TEM images of a previously reported Δ*gra7* knockout strain [31] also suggests the possibility of hyper-formation of the IVN, although additional studies are necessary to conclusively determine the magnitude and significance of this phenotype. While GRA2 and GRA6 are associated with the IVN membranes, GRA7 associates with the PVM but not directly with the IVN membranes [68]. GRA7 was previously shown to deform liposomes into tubular membranes [29], as well as interact with GRA2 and GRA6 [61]. Collectively, these observations suggest that GRA7 may coordinately regulate IVN functions in conjunction with GRA2 and GRA6. The IVN membranes were also recently implicated in heterophagy since deletion of GRA2 and subsequent loss of the IVN decreased the rate at which parasites ingest host cytosolic proteins [69]. The complex relationship of GRA protein functions in the context of PV membrane functions and IVN membrane functions merits further investigation.

The high throughput growth assay revealed significant defects in the infection rate in the Δ*gra2*, Δ*gra4*, Δ*gra5*, Δ*gra6*, Δ*gra7*, and Δ*gra9* knockout strains. However, with the exception of a slight increase in percentage of Δ*gra7* parasite vacuoles with only 1 or 2 parasites, we did not detect any significant replication rate defect in any of the single *GRA* knockout strains by direct scoring of PVs or by automated high-throughput microscopy. GRA proteins traffic as soluble protein complexes with other GRA proteins to the IVN and PVM [61]. Once inserted into the membranes they can interact with similarly localized GRA [61] and ROP proteins [30, 31]. Therefore, to determine if GRA proteins could play redundant roles, we generated double *GRA* knockout strains of GRA proteins reported to reside in GRA complexes or of GRA proteins which share similar localization: Δ*gra2*Δ*gra4*, Δ*gra2*Δ*gra6*, Δ*gra4*Δ*gra6*, Δ*gra3*Δ*gra5* and Δ*gra3*Δ*gra7*. Of these double *GRA* knockout strains, we found that the Δ*gra4*Δ*gra6*, Δ*gra3*Δ*gra5* and Δ*gra3*Δ*gra7* knockouts displayed significant replication defects *in vitro*, though the corresponding single knockouts did not. These results suggest that certain GRA proteins most likely serve redundant function(s) during the tachyzoite stage, or potentially function in a similar pathway.

Though the canonical GRA proteins are heavily expressed during tachyzoite stages [70], they appear to be dispensable for acute virulence in type I strains. None of the single or double deletion strains that we tested exhibited any significant defects in virulence following intraperitoneal infection of CD-1 mice. In contrast, several type I *GRA* knockout strains were previously reported to exhibit detectable defects in acute virulence: Δ*gra2* [42], Δ*gra6* [25, 37], and Δ*gra7* [31]. In our virulence assays we injected mice with a dose of 100 tachyzoites using the intraperitoneal route. Previously, Mercier *et*. *al* injected many mice with 10 parasites to detect a minor defect in Δ*gra2* virulence [42]. In addition, the decreased virulence phenotype of type I Δ*gra6* and Δ*gra7* mutants was previously observed following the infection of mice at atypical sites (sub-cutaneously or in the footpad) [31, 37].

Based on their pattern of secretion and their localization at the PVM as well as at the IVN, the GRA2-9 proteins may play a role in host-pathogen interactions, nutrient acquisition or vacuole integrity. While we could isolate double GRA knockouts of any double combination of Δ*gra2*, Δ*gra4*, and Δ*gra6*, we could not isolate the triple Δ*gra* knockout—Δ*gra2*Δ*gra4*Δ*gra6*. Collectively, our results suggest that while GRA2-9 individually provide non-essential functions for acute infection, some of these GRA proteins in complexes are likely to play redundant but necessary roles during acute infection. Additional experiments are still necessary to further define these functions.

The *GRA2-9* genes characterized in this study may play essential roles during transition to or growth in other life stages such as the development of tissue cysts that establish chronic infection. Most of the GRA2-9 proteins are expressed not only at the tachyzoite stage but also in the encysted bradyzoite stage [71–74]. Furthermore, a significant role for several of these GRA proteins (GRA3, GRA4, and GRA6) in type II strains has been reported [45, 46]. These findings suggest that a key role of PVM- and IVN-localized GRAs may be to prepare the PV for cyst formation or to provide essential functions during the encysted tissue stages [68]. Though the function of a large number of GRA proteins remains elusive, our study 1) has reported for the first time the *GRA3*, *GRA4*, *GRA8 and GRA9* knockouts as well as double knockouts for *GRA2-9* in the type I RH background and 2) has determined that GRA2-9 are not required for acute virulence following intraperitoneal infection of mice. Additionally we have identified that a subset of GRA proteins, which cooperate in complexes, appear to provide key functions associated with IVN formation or function.

## Supporting Information

S1 Table Primers used for generating Δ*gra* knockout strains. (DOC)

S2 Table Primers used for validating Δ*gra* knockout strains. (DOC)
