# Supplementary material for: Phenotypes Associated with Knockouts of Eight Dense Granule Gene Loci (GRA2-9) in Virulent Toxoplasma gondii
Source: PLoS One. 2016 Jul 26;11(7):e0159306. doi: 10.1371/journal.pone.0159306 (PMC4961421; doi:10.1371/journal.pone.0159306)
Supplement: S1 Table — (DOC) [file pone.0159306.s001.doc]

**S1 Table. Primers used for generating Δ*gra* knockout strains.**

| **Primer Name** | **Sequence** | **Use** | **PCR Product** |
| --- | --- | --- | --- |
| GRA1F1 | TTGGGTAACGCCAGGGTTTTCCCAGTCACGACGGTTTAAACCCTTCTGCACTGTCCGCAATCC | Construction of pΔGRA1 | 5' target |
| GRA1R1 | GCGGGTTTGAATGCAAGGTTTCGTGCTGATCAATCTAGACGCTTACGGCGTCTCATACCATG | Construction of pΔGRA1 | 5' target |
| GRA1F2 | TTCTGGCAGGCTACAGTGACACCGCGGTGGAGGTCTAGACCTGCAACTGGAAGATGCAGTGAG | Construction of pΔGRA1 | 3' target |
| GRA1R2 | GTGAGCGGATAACAATTTCACACAGGAAACAGCGCGGCCGCGCTGTTGACACAATCCGACGAGTAG | Construction of pΔGRA1 | 3' target |
| GRA2F1 | TTGGGTAACGCCAGGGTTTTCCCAGTCACGACGGTTTAAACCTATGGAACAAGCCGTGTGTCGATAG | Construction of pΔGRA2 | 5' target |
| GRA2R1 | GCGGGTTTGAATGCAAGGTTTCGTGCTGATCAAACTAGTTCGCACTTAGCCTAAGGGACGAC | Construction of pΔGRA2 | 5' target |
| GRA2F2 | TTCTGGCAGGCTACAGTGACACCGCGGTGGAGGACTAGTTGGAGAGTGACAGAAGCACTACGAC | Construction of pΔGRA2 | 3' target |
| GRA2R2 | GTGAGCGGATAACAATTTCACACAGGAAACAGCGCGGCCGCCGCCCTCGTGATTCCTCCAAG | Construction of pΔGRA2 | 3' target |
| GRA3F1 | TTGGGTAACGCCAGGGTTTTCCCAGTCACGACGGTTTAAACCATAACAACCCAGGTCTCGCGTC | Construction of pΔGRA3 | 5' target |
| GRA3R1 | GCGGGTTTGAATGCAAGGTTTCGTGCTGATCAAACTAGTCGATCCTTGCAGGACATCTCGTC | Construction of pΔGRA3 | 5' target |
| GRA3F2 | TTCTGGCAGGCTACAGTGACACCGCGGTGGAGGACTAGTGAGGCAACCCTTCATGAGTTCGG | Construction of pΔGRA3 | 3' target |
| GRA3R2 | GTGAGCGGATAACAATTTCACACAGGAAACAGCCTCGAGCGAGCAGCGATGTAGTTCACGATC | Construction of pΔGRA3 | 3' target |
| GRA4F1 | TTGGGTAACGCCAGGGTTTTCCCAGTCACGACGGTTTAAACCCTCAGCGGTTATCCTAACGTCCA | Construction of pΔGRA4 | 5' target |
| GRA4R1 | GCGGGTTTGAATGCAAGGTTTCGTGCTGATCAAACTAGTCATCTGTACCTGCTTGCGTGGAG | Construction of pΔGRA4 | 5' target |
| GRA4F2 | TTCTGGCAGGCTACAGTGACACCGCGGTGGAGGACTAGTCGGAGTTGGACGACGGATATCG | Construction of pΔGRA4 | 3' target |
| GRA4R2 | GTGAGCGGATAACAATTTCACACAGGAAACAGCCTCGAGGTCTATCTTGTCACTCCGGACGGTA | Construction of pΔGRA4 | 3' target |
| GRA5F1 | TTGGGTAACGCCAGGGTTTTCCCAGTCACGACGGTTTAAACCATCACTGCTGTCCACTGTAGTGC | Construction of pΔGRA5 | 5' target |
| GRA5R1 | GCGGGTTTGAATGCAAGGTTTCGTGCTGATCAAACTAGTCAGCCCACACGTCTGATTCCAC | Construction of pΔGRA5 | 5' target |
| GRA5F2 | TTCTGGCAGGCTACAGTGACACCGCGGTGGAGGACTAGTCTGGACATCCAGTGAGGACTGCA | Construction of pΔGRA5 | 3' target |
| GRA5R2 | GTGAGCGGATAACAATTTCACACAGGAAACAGCGCGGCCGCAGTTACGCGTACATAGACCTGCAGG | Construction of pΔGRA5 | 3' target |
| GRA6F1 | TTGGGTAACGCCAGGGTTTTCCCAGTCACGACGGTTTAAACTAGATACCGGGATCGATCATCGCAC | Construction of pΔGRA6 | 5' target |
| GRA6R1 | GCGGGTTTGAATGCAAGGTTTCGTGCTGATCAAACTAGTCGGTGGAACAAGATGAGAAGCATCC | Construction of pΔGRA6 | 5' target |
| GRA6F2 | TTCTGGCAGGCTACAGTGACACCGCGGTGGAGGACTAGTCTGGGAACGGTGGGAATGAAGG | Construction of pΔGRA6 | 3' target |
| GRA6R2 | GTGAGCGGATAACAATTTCACACAGGAAACAGCGCGGCCGCCTCAACGCATGTAGGTGTCGCTG | Construction of pΔGRA6 | 3' target |
| GRA7F1 | TTGGGTAACGCCAGGGTTTTCCCAGTCACGACGGTTTAAACCGTGCATCCAGAACCTTCTGTCC | Construction of pΔGRA7 | 5' target |
| GRA7R1 | GCGGGTTTGAATGCAAGGTTTCGTGCTGATCAAACTAGTCGGTAGCGACGAATGGATACTGGA | Construction of pΔGRA7 | 5' target |
| GRA7F2 | TTCTGGCAGGCTACAGTGACACCGCGGTGGAGGACTAGTGAGGTGCCTGAATCAGGCGAAG | Construction of pΔGRA7 | 3' target |
| GRA7R2 | GTGAGCGGATAACAATTTCACACAGGAAACAGCGCGGCCGCATCGAGTGCTCTCTACTGCCACCTA | Construction of pΔGRA7 | 3' target |
| GRA8F1 | TTGGGTAACGCCAGGGTTTTCCCAGTCACGACGGTTTAAACGTCTACGCTGCTATCTTGCATCCCA | Construction of pΔGRA8 | 5' target |
| GRA8R1 | GCGGGTTTGAATGCAAGGTTTCGTGCTGATCAAACTAGTCAGGAGGTATCCCTCGAGACGTT | Construction of pΔGRA8 | 5' target |
| GRA8F2 | TTCTGGCAGGCTACAGTGACACCGCGGTGGAGGACTAGTCAATGGCAGCAGCCTTGATAACCC | Construction of pΔGRA8 | 3' target |
| GRA8R2 | GTGAGCGGATAACAATTTCACACAGGAAACAGCGCGGCCGCGTGTTGTTCCTGACCTCAGCATGG | Construction of pΔGRA8 | 3' target |
| GRA9F1 | TTGGGTAACGCCAGGGTTTTCCCAGTCACGACGGTTTAAACGATCCGGAGTTACACAGCACCCT | Construction of pΔGRA9 | 5' target |
| GRA9R1 | GCGGGTTTGAATGCAAGGTTTCGTGCTGATCAAACTAGTCGGCTTATCGGGACGACTCTCTT | Construction of pΔGRA9 | 5' target |
| GRA9F2 | TTCTGGCAGGCTACAGTGACACCGCGGTGGAGGACTAGTCCAAGAGGTCGAACTCGAGGAGAA | Construction of pΔGRA9 | 3' target |
| GRA9R2 | GTGAGCGGATAACAATTTCACACAGGAAACAGCGCGGCCGCTTAGGCTGGCAAGCTCTCCCTAC | Construction of pΔGRA9 | 3' target |
| GRA10F1 | TTGGGTAACGCCAGGGTTTTCCCAGTCACGACGGTTTAAACGTACTCCAAGGTCGTGTTGCAGC | Construction of pΔGRA10 | 5' target |
| GRA10R1 | GCGGGTTTGAATGCAAGGTTTCGTGCTGATCAAACTAGTGATATCCTGTACACGCCGAACGC | Construction of pΔGRA10 | 5' target |
| GRA10F2 | TTCTGGCAGGCTACAGTGACACCGCGGTGGAGGACTAGTCAATTCTGTCATCGAAACGTGCAGC | Construction of pΔGRA10 | 3' target |
| GRA10R2 | GTGAGCGGATAACAATTTCACACAGGAAACAGCGCGGCCGCTACAGTAGCCATCGCCCGTCAG | Construction of pΔGRA10 | 3' target |
|  |  |  |  |
