# Supplementary material for: Phenotypes Associated with Knockouts of Eight Dense Granule Gene Loci (GRA2-9) in Virulent Toxoplasma gondii
Source: PLoS One. 2016 Jul 26;11(7):e0159306. doi: 10.1371/journal.pone.0159306 (PMC4961421; doi:10.1371/journal.pone.0159306)
Supplement: S2 Table — (DOC) [file pone.0159306.s002.doc]

**S2 Table. Primers used for validating Δ*gra* knockout strains.**

| Primer Name | Sequence | Use | PCR Product |
| --- | --- | --- | --- |
| **GRA1CxF** | CTAGAACACCTGCTGCGTGCAG | Validation of RH*Δku80Δgra1::HXGPRT* | PCR3 |
| **GRA1CxR** | TAGCCACGCTTACGTATTACACCTG | Validation of RH*Δku80Δgra1::HXGPRT* | PCR2/PCR4 |
| **GRA1ExF** | GTTCTCCAGTCCTTTCTCAGTGACG | Validation of RH*Δku80Δgra1::HXGPRT* | PCR2 |
| **GRA1ΔF** | GTGAGCGCTATTGTCGGAGCTG | Validation of RH*Δku80Δgra1::HXGPRT* | PCR1 |
| **GRA1ΔR** | GAAGAACGTGCTTCACTCCACTCC | Validation of RH*Δku80Δgra1::HXGPRT* | PCR1 |
| **GRA2CxF** | GTTAACAGTTCCCTTGTGGCTGGTC | Validation of RH*Δku80Δgra2::HXGPRT* | PCR3 |
| **GRA2CxR** | CCCCAGGATAATGCAGAAAAGCTGG | Validation of RH*Δku80Δgra2::HXGPRT* | PCR2/PCR4/PCR5 |
| **GRA2ExF** | GACGCTCCCAGATGTCATATGCC | Validation of RH*Δku80Δgra2::HXGPRT* | PCR2 |
| **GRA2ΔF** | GGTGAACATACACCACCACTCCC | Validation of RH*Δku80Δgra2::HXGPRT* | PCR1 |
| **GRA2ΔR** | CTGGAACCTCGGTTTCTGCCAG | Validation of RH*Δku80Δgra2::HXGPRT* | PCR1 |
| **GRA2cF** | GATACTGACTGGTGACTTGCACGTAC | Validation of RH*Δku80Δgra2Δhxgprt* | PCR5 |
| **GRA3CxF** | GCAAGGTCAACAGGGGTCCTTC | Validation of RH*Δku80Δgra3::HXGPRT* | PCR3 |
| **GRA3CxR** | TCCGGACGTGTGTCCTGAGAG | Validation of RH*Δku80Δgra3::HXGPRT* | PCR2/PCR4/PCR5 |
| **GRA3ExF** | TTTGCCTCATTTGTGTACCGTCGTG | Validation of RH*Δku80Δgra3::HXGPRT* | PCR2 |
| **GRA3ΔF** | AATCATCAGGCTCTTGCAGAACCAG | Validation of RH*Δku80Δgra3::HXGPRT* | PCR1 |
| **GRA3ΔR** | GTATATTGTCTCGCAACGTCCTGGAA | Validation of RH*Δku80Δgra3::HXGPRT* | PCR1 |
| **GRA3cF** | GAACGCTCTTGGGATCCGTAATGC | Validation of RH*Δku80Δgra3Δhxgprt* | PCR5 |
| **GRA4CxF** | CGTGCCCTCGCACTTTAGTCG | Validation of RH*Δku80Δgra4::HXGPRT* | PCR3 |
| **GRA4CxR** | CTGTCCCTGGTTTGCAAGTAAGCAC | Validation of RH*Δku80Δgra4::HXGPRT* | PCR2/PCR4/PCR5 |
| **GRA4ExF** | GAGCAGTGTATGTGGCTGACTTCTG | Validation of RH*Δku80Δgra4::HXGPRT* | PCR2 |
| **GRA4ΔF** | CCCTACGCAAATGGACAGCAGG | Validation of RH*Δku80Δgra4::HXGPRT* | PCR1 |
| **GRA4ΔR** | GACTGTTGTCTCAACTGCAGACCAG | Validation of RH*Δku80Δgra4::HXGPRT* | PCR1 |
| **GRA4cF** | TTCGTAGCTAGCATTCTCTAATCATCGC | Validation of RH*Δku80Δgra4Δhxgprt* | PCR5 |
| **GRA5CxF** | GACCTTATCGTGTACTGGTGCAAGC | Validation of RH*Δku80Δgra5::HXGPRT* | PCR3 |
| **GRA5CxR** | TCCTCTAGTACCGTCTCTCATGTGC | Validation of RH*Δku80Δgra5::HXGPRT* | PCR2/PCR4 |
| **GRA5ExF** | TTGCGTGATGATCGTTGTGGTGTAG | Validation of RH*Δku80Δgra5::HXGPRT* | PCR2 |
| **GRA5ΔF** | GTAATGATCGTGAACGTGCTGGCTT | Validation of RH*Δku80Δgra5::HXGPRT* | PCR1 |
| **GRA5ΔR** | TCCTCGGCAACTTCTTCCTCTTCC | Validation of RH*Δku80Δgra5::HXGPRT* | PCR1 |
| **GRA6CxF** | TCTGTGTCGCAGACATGTCTACCC | Validation of RH*Δku80Δgra6::HXGPRT* | PCR3 |
| **GRA6CxR** | ACCGTCTGCACTCTTCTGTAGGG | Validation of RH*Δku80Δgra6::HXGPRT* | PCR2/PCR4 |
| **GRA6ExF** | CGGTGAAGCCGAATCTGTCGAG | Validation of RH*Δku80Δgra6::HXGPRT* | PCR2 |
| **GRA6ΔF** | CAGCTTCGTGGTGCCACGTAG | Validation of RH*Δku80Δgra6::HXGPRT* | PCR1 |
| **GRA6ΔR** | GCTTCGGAGGTTGTATCATCTTCAGC | Validation of RH*Δku80Δgra6::HXGPRT* | PCR1 |
| **GRA7CxF** | ACTCGTGGTTCTCCCCTGTGAG | Validation of RH*Δku80Δgra7::HXGPRT* | PCR3 |
| **GRA7CxR** | CTCGAATGTGTCAGCTGGATTGCAG | Validation of RH*Δku80Δgra7::HXGPRT* | PCR2/PCR4 |
| **GRA7ExF** | CCTGGCAGCATCACGTAGTGG | Validation of RH*Δku80Δgra7::HXGPRT* | PCR2 |
| **GRA7ΔF** | ATGACGAACTGATGAGTCGAATCCG | Validation of RH*Δku80Δgra7::HXGPRT* | PCR1 |
| **GRA7ΔR** | AAATACGATGCACCCATACCAACAGC | Validation of RH*Δku80Δgra7::HXGPRT* | PCR1 |
| **GRA8CxF** | CTGGACGCTACCGAACTCGC | Validation of RH*Δku80Δgra8::HXGPRT* | PCR3 |
| **GRA8CxR** | CTAACGTGTCGGTCGTGCATGTC | Validation of RH*Δku80Δgra8::HXGPRT* | PCR2/PCR4 |
| **GRA8ExF** | CGTGCTAACGTCCTGAGAAACGC | Validation of RH*Δku80Δgra8::HXGPRT* | PCR2 |
| **GRA8ΔF** | CATCCAAGCAGTTACGGAGCGTC | Validation of RH*Δku80Δgra8::HXGPRT* | PCR1 |
| **GRA8ΔR** | ATGGTGAACTGCCGGTATCTCCG | Validation of RH*Δku80Δgra8::HXGPRT* | PCR1 |
| **GRA9CxF** | CTACGCCCTGGTGAACACTAGAC | Validation of RH*Δku80Δgra9::HXGPRT* | PCR3 |
| **GRA9CxR** | CAACACTGAATCTGAATGGAAACGCG | Validation of RH*Δku80Δgra9::HXGPRT* | PCR2/PCR4 |
| **GRA9ExF** | CTGTGTCGTGAGCGAGTGACTTC | Validation of RH*Δku80Δgra9::HXGPRT* | PCR2 |
| **GRA9ΔF** | CTACTCGCCGTGATGAGAAGACG | Validation of RH*Δku80Δgra9::HXGPRT* | PCR1 |
| **GRA9ΔR** | GACGTCACAGGGCTGGAATCC | Validation of RH*Δku80Δgra9::HXGPRT* | PCR1 |
| **GRA10CxF** | GGACGAGAACCAGATGATCCACG | Validation of RH*Δku80Δgra10::HXGPRT* | PCR3 |
| **GRA10CxR** | ATCTGTATGGAGCTGGGACAGACG | Validation of RH*Δku80Δgra10::HXGPRT* | PCR2/PCR4 |
| **GRA10ExF** | GCGTGTGCATATATACAAGCGTCTGC | Validation of RH*Δku80Δgra10::HXGPRT* | PCR2 |
| **GRA10ΔF** | CTTCTGCAGCTGCTGCAACTCC | Validation of RH*Δku80Δgra10::HXGPRT* | PCR1 |
| **GRA10ΔR** | GGGAAGCAACGCACAGTACCC | Validation of RH*Δku80Δgra10::HXGPRT* | PCR1 |
| **HX.F** | GATAAGCTTGATCAGCACGAAACCTTG | Validation of RH*Δku80Δgoi::HXGPRT* | PCR4 |
| **HX.R** | CCGCTCTAGAACTAGTGGATCCC | Validation of RH*Δku80Δgoi::HXGPRT* | PCR3 |
